# Degradation of naturally produced hydroxylated polybrominated diphenyl ethers in Baltic Sea sediment via reductive debromination

**DOI:** 10.1007/s11356-021-12462-3

**Published:** 2021-01-21

**Authors:** Dennis Lindqvist, Johan Gustafsson

**Affiliations:** Department of Environmental science, Stockholm University, SE-106 91 Stockholm, Sweden

**Keywords:** Reduction, OH-PBDE, PBDE, Biodegradation, Algae, Sediment

## Abstract

Over the last two decades, the occurrence of hydroxylated polybrominated diphenyl ethers (OH-PBDEs) has been observed to be nearly ubiquitous among Baltic Sea filamentous macroalgae. High concentrations are continuously recorded among red, green, and brown filamentous algae. Several of these algae species are ephemeral, and when large parts of the colonies decay at the end of their lifecycles, the OH-PBDEs are expected to largely partition to the sediment. In this study, the fate of OH-PBDEs in Baltic Sea sediment was investigated, with focus on the effect of reductive debromination. During chemical debromination, it was observed that the half-life could differ with as much as two orders of magnitude between a pentabrominated and a tetrabrominated congener. Using collected Baltic Sea sediment, it was further observed that the half-life of spiked pentabrominated OH-PBDEs spanned from a few days up to a few weeks in room temperature. At 4 °C, it took 6 months to achieve a 50% decrease in concentration of the fasted degrading congener. Clear differences in selectivity between chemical debromination and debromination in sediment were also observed when studying the major reaction products. Baltic Sea sediment seems to have a good capacity for reducing naturally produced OH-PBDEs.

**Supplementary Information:**

The online version contains supplementary material available at 10.1007/s11356-021-12462-3.

## Introduction

Many halogenated organic compounds are known to be naturally produced in both the terrestrial and marine environments, by, for example, algae, fungi, sponges, and bacteria (Gribble 2010). In the Baltic Sea, brominated substance such as bromophenols, hydroxylated polybrominated diphenyl ethers (OH-PBDEs), methoxylated PBDEs (MeO-PBDEs), and polybrominated dibenzo-*p*-dioxins have been identified in several species of filamentous macroalgae (Löfstrand et al. 2011). During summer in the Baltic Sea, the biomass of several species of filamentous macroalgae increases (HELCOM 2009). Furthermore, a large increase in the production of OH-PBDEs has also been observed during the summer, in the red algae *Ceramium tenuicorne* (Dahlgren et al. 2015). The increased biomass of filamentous macroalgae together with increased production of OH-PBDEs within these algae leads to a large input of OH-PBDEs to the Baltic Sea during summer. The large input in turn results in an increased exposure to OH-PBDEs among Baltic wildlife, as observed in, for example, blue mussels (Löfstrand et al. 2011). The concentration of these compounds in blue mussels has been observed to be much larger than the concentration of anthropogenic brominated pollutants, specifically the brominated flame retardant BDE47 (Löfstrand et al. 2011). Exposure to OH-PBDEs in wildlife may also stem from accumulation of algae produced MeO-PBDEs and a subsequent metabolic demethylation (Wan et al. 2009).

Upon death and decomposition of OH-PBDE-producing algae in the Baltic Sea, large quantities of OH-PBDEs are expected to be transferred to the sediment. High sorption to sediment has been reported for some chlorinated phenols (Isaacson and Frink 1984). Sorption to sediment does not only occur in the less polar protonated form but also in the ionic form (Schellenberg et al. 1984), which is important for OH-PBDEs that generally have p*K*_*a*_ values close to or below the pH of seawater. Thus, sediment is expected to be the final sink for a large proportion of the naturally produced brominated compounds in the Baltic Sea. Based on the high concentrations of OH-PBDEs detected in *C. tenuicorne* (Dahlgren et al. 2015; Lindqvist et al. 2017) as well as in other macroalgae in the Baltic Sea (Löfstrand 2011), the annual input of OH-PBDEs to Baltic Sea sediment is expected to be large.

The structurally related compound decabromodiphenyl ether (BDE209) has previously been shown to undergo reductive debromination in anaerobic sediment, and a large variety of lesser brominated PBDEs were observed to be produced (Tokarz et al. 2008). When Zhang and co-workers (2012) studied the transformation of 6-OH-BDE47 in Chinese sediment, a methylation to 6-MeO-BDE47 was observed.

The present study was divided into two parts. The first part was aimed to assess the relative chemical stability of the most abundant naturally produced OH-PBDE congeners in the Baltic Sea towards reductive debromination, while simultaneously creating a map over degradation products formed by each congener. The second part was aimed to investigate the degradation of OH-PBDEs in collected Baltic Sea sediment and the role reductive debromination has in any observed degradation.

## Materials and methods

### Chemicals and reagents

All solvents used were of analytical grade quality and purchased from established brands. Metallic zinc powder was purchased from Sigma-Aldrich (Steinheim, Germany). All OH-PBDEs and MeO-PBDEs used for degradation tests and as reference compounds for sediment analysis were synthesized in house by Marsh and co-workers (2003). BDE99 and BDE139 were purchased from Wellington Laboratories (Guelph, ON, Canada). Diazomethane used for derivatization of OH-PBDEs was synthesized in house from *N*-methyl-*N*-nitroso-*p*-toulenesulfonamide (Diazald) purchased from Sigma-Aldrich (Steinheim, Germany).

### Samples

Sediment samples were collected in late June outside of Askö, Sweden (Lat. 58.8215, Long. 17.6305), using an Ekman grab sampler and were stored at 4 °C; seawater was collected simultaneously. Macroscopic objects such as algae and mussels were removed by hand from the sediment before centrifugation, decanting of the water, and mixing of the sediment by hand. The water sample was filtered through a filter paper followed by a 0.22-μm membrane filter (Millipore, Burlington, MA, USA). The experiments were started the day after the samples were collected.

### Chemical reductive debromination

Chemical debromination was conducted in a similar manner as one of the methods presented for reduction of 2,4,6-tribromophenol by Tashiro et al. (1976). Nine OH-PBDEs were degraded, including one hexabrominated congener: 6-OH-BDE137; four pentabrominated congeners: 6-OH-BDE85, 2-OH-BDE123, 6-OH-BDE90, and 6-OH-BDE99; and four tetrabrominated congeners: 2′-OH-BDE66, 2′-OH-BDE68, 6′-OH-BDE49, and 6-OH-BDE47. Two neutral pentabrominated compounds were also included in the experiment: 6-MeO-BDE99 and BDE99. Each test substance (1 μg) dissolved in ethanol (1.2 mL) was added to a 2-mL Eppendorf tube containing zinc powder (50 mg). Hydrochloric acid (HCl; 1.5 M, 0.3 mL) was added, and the sample was incubated at 50 °C on an Eppendorf Thermomixer comfort (Eppendorf, Hamburg, Germany) mixing at 750 rpm. All compounds were tested individually in triplicates. Fifty microliters of the test solution was taken out at different time points and was transferred to a new Eppendorf tube containing water (100 *μ*L) and either pentane/diethyl ether (9:1, 1.2 mL) for OH-PBDEs or 2,2,4-trimethylpentane (TMP; 0.7 mL) for PBDEs and MeO-PBDEs. The samples were mixed and the organic phase isolated. Neutral compounds were analyzed directly on gas chromatography mass spectrometry (GC-MS) whiles the OH-PBDEs were methylated using ethereal diazomethane (300 *μ*L) for 1.5 h at ambient temperature in darkness. After methylation, the samples were evaporated to dryness and resolved in TMP (0.7 mL) before analysis.

For the hexabrominated 6-OH-BDE137, aliquot samples were taken after 0, 1, 3, 6, 10, and 15 min. For pentabrominated OH-PBDEs and MeO-BDE99, aliquot samples were taken after 0, 2, 5, 9, 14, and 20 min. For BDE99, aliquots were taken at 0, 5, 15, 30, and 60 min. For 2′-OH-BDE66 and 2′-OH-BDE68, aliquots were taken at 0, 15, 45, 90, and 150 min. Lastly, for 6-OH-BDE47 and 6′-OH-BDE49, aliquot samples were taken at 0, 45, 90, 180, and 240 min. The variation in sampling time points depended on the varying reaction speed of each congener. Zinc and HCl were used in excess to achieve a pseudo first-order reaction. However, after 1 h, the reaction started to become limited by the acid. Hence, an additional drop of HCl (6 M) was added to maintain first-order kinetics when aliquot samples were taken out for the tetrabrominated OH-PBDEs, after 45 and 90 min for 2′-OH-BDE66 and 2′-OH-BDE68, and after 45, 90, and 180 min for 6-OH-BDE47 and 6′-OH-BDE49. Increasing the amount of acid in the beginning of the reaction was avoided, to avoid high pressure build up in the Eppendorf tubes.

### Degradation of OH-PBDEs in sediment

A standard mixture composed of 6-OH-BDE90, 6-OH-BDE99, 2-OH-BDE123, and 6-OH-BDE85, 100 ng (170 pmol) each in 100 μL of methanol, was spiked into a sterile test tube. The methanol was evaporated of until only a droplet remained before adding 1 g of sediment and 4 mL of filtered seawater. The ratio of sediment to water was the same as in the study conducted by Zhang and co-workers (2012). The test tubes, 27 in total, were placed on a tipping board during 48 h to achieve equilibrium. Three samples were then analyzed to determine the starting concentrations of OH-PBDEs and MeO-PBDEs in the sediment. The remaining samples were stored in darkness, at either room temperature or at 4 °C. Three separate samples were analyzed per time point. The samples stored at room temperature were taken for analyses after 7, 30, 90, and 180 days respectively (in total 12 samples) while the samples stored at 4 °C were taken for analyses after 7, 30, 180, and 360 days respectively (in total 12 samples). Before analysis, all samples were centrifuged at 2000 *g* for 5 min and the water was decanted off. 4′-OH-BDE121 (50 ng) and BDE139 (5 ng) were added as surrogate internal standards for phenolic and neutral compounds, respectively.

### Determination of native levels of OH-PBDEs

Two 5 g samples of sediment were analyzed to quantify the native occurrence of OH-PBDEs and MeO-PBDEs. 4′-OH-BDE121 (5 ng) and BDE139 (1 ng) were added as internal standards for phenolic and neutral compounds, respectively, before starting the extraction. BDE139 (5 ng) was also added as volumetric standard in the phenolic fraction prior to instrumental analysis to calculate the recovery of the surrogate. A procedural blank sample was run in parallel to the samples, and instrumental blanks were run before and after all analyses; all analytes were below the limit of quantification (LOQ; 5 times the level of the surrounding noise) in the blanks. Only trace amounts of 6-OH-BDE47 and 6-OH-BDE85 could tentatively be detected in the instrumental blanks, but not in any levels that could influence the results; hence, no blank subtraction was needed.

### Extraction of sediment

The extractions were performed using a slightly modified version of the procedure described by Lindqvist and Asplund (2019). Briefly, to 1 g of sediment acetonitrile/2-propanol (3 : 1, 4 mL) with 1% acetic acid was added before mixing the sample on a vortex mixer. The sample was then mixed again following the addition of diethyl ether (DEE; 2 mL) before centrifuging the sample at 2000 *g* for 5 min. The supernatant was transferred to a new test tube, and the sediment pellet was re-extracted with 2-methylpentane/diethyl ether (IHX/DEE; 3 : 1, 3 mL). The sample was centrifuged and the supernatants from the two extractions combined. HCl (6 M, 0.5 mL), water (4 mL), and IHX (3 mL) were added to the extract. After mixing and centrifugation, the organic phase was transferred to a new test tube (for the 5 g samples the extraction method was scaled up accordingly). The volume was reduced to approximately 4 mL under a stream of nitrogen before adding potassium hydroxide (0.5 M, 3 mL) in water/ethanol (1 : 1). After mixing and centrifugation, the aqueous phase (phenolic fraction) and organic phase (neutral fraction) were transferred to separate test tubes.

The phenolic fraction was acidified with HCl (6 M, 0.5 mL) and the phenolic compounds were back-extracted into IHX (3 mL). The aqueous phase was re-extracted with an additional portion of IHX (1 mL). After combining the organic phases, the volume was reduced to 0.5 mL and a few drops of methanol were added. Derivatization was performed by adding ethereal diazomethane (300 μL). The reaction was allowed to proceed for 1.5 h at ambient temperature in darkness before the excess diazomethane was evaporated off under a gentle stream of nitrogen gas. After adjusting the volume to 3 mL with IHX, concentrated sulfuric acid (1.5 mL) was added. The sample was mixed and centrifuged before the IHX was transferred to a new test tube. The sulfuric acid was re-extracted with 2,2,4-trimethylpentane (TMP; 1 mL).

The neutral fraction was reduced to 3 mL and treated with sulfuric acid in the same manner as the phenolic fraction. The neutral fraction was then further reduced to 2 mL, and sulfur was removed by shaking the sample (in a closed test tube) under a tap of streaming hot water for 1 min with 2-propanol (2 mL) and a tetrabutylammonium hydrogen sulfate aqueous solution (4% w/w) saturated with sodium sulfite (2 mL), according to Nylund and co-workers (1992). Water (5 mL) and TMP (1 mL) was added to the sample, and after phase separation by centrifugation, the organic phase was isolated. Both the neutral and the phenolic fractions were reduced by nitrogen gas until only TMP remained, and the volumes were further adjusted to proper levels before analysis on GC-MS.

### Instrumental analysis

Analyses were conducted on an Agilent 7890A GC (Agilent Technologies, CA, USA) with a multimode injector (MMI), coupled to a 5975C MS. All analyses were done in the electron capture negative ionization mode (ECNI). A 5-μL aliquot was injected in liquid vent mode at 75 °C. The solvent was vented at 5 psi for 0.04 min. The injector was then ramped in two stages: first, to 255 °C (held for 1.86 min), and then, to 325 °C, both times at 600 °C/min. The split was opened after 2.2 min with a purge flow of 60 mL/min. Helium was used as carrier gas with a flow of 1.25 mL/min. The GC oven was programmed from 65 °C (held for 2.2 min) to 250 °C at 25 °C/min, then to 285 at 5 °C/min, and finally to 310 °C at 25 °C/min (held for 5.4 min). The whole program was 23 min. A J&W DB-35MS UI capillary column was used (20 m × 0.18 mm i.d. × 0.18 μm film thickness; J&W Scientific, CA, USA). The transfer line to the MS was set at 310 °C, the ion source temperature was 200 °C, and the quadrupole temperature was 150 °C. Methane was used as buffer gas for ECNI/MS, with a vacuum pressure of ~1.9E-04 Torr. Quantifications of OH-PBDEs were conducted using selected ion monitoring (SIM) of the bromide ions *m/z*: 79, 81. All quantifications were conducted using 5-point external calibration curves with authentic reference standards.

## Result and discussion

### Chemical reductive debromination

**Fig. 1:**
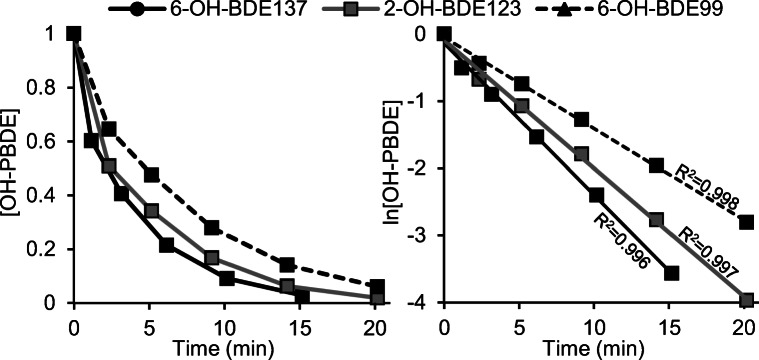
Example of the chemical reductive debromination of OH-PBDEs displaying the degradation for three congeners. Average values from three experiments. Full data set can be found in the electronic supplementary material (ESM), Table S1

During chemical debromination, all test compounds degraded according to first-order kinetics (see example in Fig. 1). The rate of debromination was affected both by the level of oxidation (amount of bromines) as well as the substitution pattern, particularly by the occurrence of sterically congested bromines (i.e., bromines with two adjacent bulky substituents, e.g., Br/Br or Br/OH). The difference in half-lives (*t*_1/2_) between the pentabrominated congeners and the two slowest degrading tetrabrominated congeners (6-OH-BDE47 and 6′-OH-BDE49) was as large as 2 orders of magnitude (see Table 1). In fact, 6-OH-BDE47 and 6′-OH-BDE49 did not reach *t*_1/2_ within the time span of the experiment and their recorded *t*_1/2_ values in Table 1 should only be considered tentative.

**Table 1:** Chemical debromination rates provided as t_1/2_ (min) ± one standard deviation. All experiments were made in triplicates

Br_*n*_	Congener	*t*_1/2_ (min)		
6	6-OH-BDE137	3.1	±	0.06
5	6-OH-BDE85	3.5	±	0.5
5	2-OH-BDE123	3.6	±	0.5
5	6-OH-BDE90	4.6	±	0.6
5	6-OH-BDE99	5.1	±	0.7
5	6-MeO-BDE99	4.4	±	0.2
5	BDE99	9.7	±	0.3
4	2′-OH-BDE66	23	±	3.0
4	2′-OH-BDE68	122	±	14
4	6′-OH-BDE49	364^A^	±	75
4	6-OH-BDE47	417^A^	±	28

^A^These values should be considered approximations

The effect of sterically congested bromines on the reduction rate can be observed by comparing the *t*_1/2_ for the different tetrabrominated OH-PBDEs in Table 1. The only tetrabrominated OH-PBDE in Table 1 that has a sterically congested bromine is 2′-OH-BDE66, and this congener degraded 5 times faster than the second fastest degrading tetrabrominated congener. No statistically significant difference could be observed between 6-OH-BDE99 and 6-MeOBDE99 in this system, while both showed statistical difference compared to BDE99 (using 2 sided *t*-tests, *p* < 0.05). BDE99 may differ from its methoxylated and hydroxylated counterparts both because it is less oxidized (it lacks the additional oxygen from the MeO/OH group) and because it lacks sterically congested bromines.

**Fig. 2:**
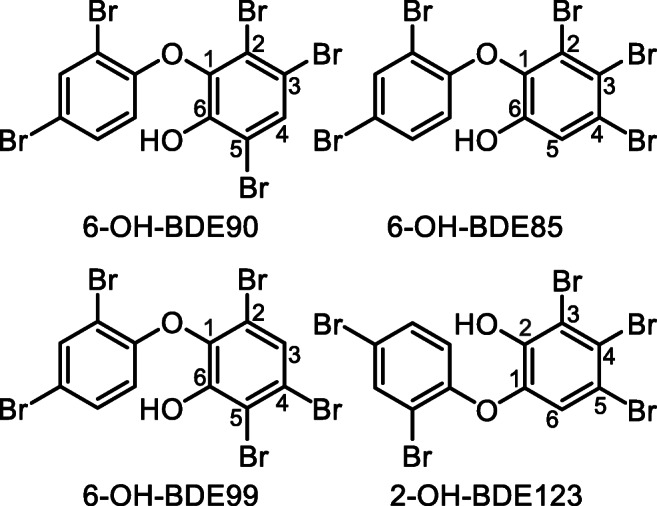
Chemical structures of the pentabrominated OH-PBDEs included in this study. The structures of all tetrabrominated OH-PBDEs that can be formed by removal of one Br from each of the four compounds can be found in the ESM, Fig. S1

The inclination of sterically congested bromines to be removed first can also be observed in the products formed when degrading the pentabrominated OH-PBDEs. 6-OH-BDE90 and 6-OH-BDE99 have one sterically congested bromine each (in position 2 and 5, respectively, see Fig. 2), and both degrade to form one major product by loss of their, respectively, sterically congested bromine. 6-OH-BDE90 form 2′-OH-BDE68, and 6-OH-BDE99 form 6-OH-BDE47. Both congeners also form 6′-OH-BDE49 as a moderate to minor product (via loss of the Br in position 3 and 4, respectively, see Fig. 2). 6-OH-BDE99 also forms 2′-OH-BDE66 as well as an unknown congener as minor products (see Table S2 in the ESM). 6-OH-BDE85 and 2-OH-BDE123 on the other hand has two sterically congested bromines (in position 2 and 3, respectively, 3 and 4, see Fig. 2) and hence form two major products. 6-OH-BDE85 form 6′-OH-BDE66 (by loss of Br in position 2) and 6-OH-BDE47 (by loss of Br in position 3), while 2-OH-BDE123 form 6′-OH-BDE66 (by loss of Br in position 3) and 2′-OH-BDE68 (by loss of Br in position 4) (see Fig. 2). Both also form minor products (see Table S2 in the ESM). The exact formation rate was not measured as the products themselves start to degrade at variable speed upon formation resulting in the formation of tribrominated congeners (see Table S2 in the ESM). The steric congestion likely leads to tension in the molecule due to unfavorable bond angles, which in turn results in a higher energy state. Removal of the sterically congested bromines would relieve the tension leading to the lowest energy product. The dominant formation of 6-OH-BDE47, 2′-OH-BDE68, and 6′-OH-BDE66 from the four pentabrominated congeners shown in Fig. 2 can thus be predicted by calculating the energies of the products formed, see Table S3 in the ESM.

The degradation rate of 6′-OH-BDE66 was not measured due to the lack of a pure standard during the experiments (6′-MeO-BDE66 was however available in a mix of MeO-PBDEs for identification of methylated 6’-OH-BDE66). The lack of sterically congested bromines in 6′-OH-BDE66 does however suggest that it at least would degrade slower than 2′-OH-BDE66. Only two tribrominated congener (6′-OH-BDE17 and 2′-OH-BDE28) were available as standards for positive identification among the products formed by debromination of the tetrabrominated OH-PBDEs. However, a full list of the different products that were detected, together with their respective retention time on the GC system used, can be found in the ESM, Table S2.

### Degradation of OH-PBDEs in sediment

**Fig. 3:**
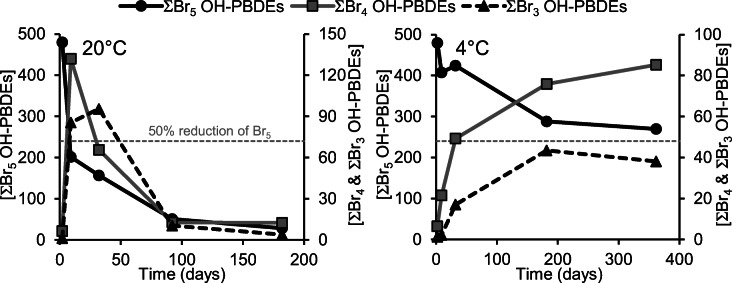
Degradation of spiked pentabrominated OH-PBDEs in sediment and formation of the major tetra- and tribrominated congeners, at room temperature as well as at 4 °C. Concentrations are provided in pmol/g wet weight. Raw data can be found in the ESM, Table S4 and S5

The four pentabrominated OH-PBDEs chosen for this experiment are the dominant pentabrominated congeners detected in Baltic Sea algae, as well as in other Baltic Sea organisms (Löfstrand 2011; Lindqvist 2016). All four OH-PBDEs were observed to degrade rapidly in the spiked sediment at room temperature (see Fig. 3). 6-OH-BDE90 and 6-OH-BDE99 degraded by more than 50% within the first 7 days (see Table S4 in the ESM). In direct contrast to the results from the chemical debromination, 6-OH-BDE90 and 6-OH-BDE99 were observed to degrade faster than 2-OH-BDE123 and 6-OH-BDE85 in sediment. 2-OH-BDE123 degraded only slightly slower, while 6-OH-BDE85 degraded significantly slower than 6-OH-BDE90 and 6-OH-BDE99 (see Table S4 in the ESM).

The major debromination products formed also differed from that obtained during the chemical debromination experiment. The microbial debromination of the four pentabrominated OH-PBDEs in sediment initially yielded a quite large diversity of tetrabrominated congeners. In total, eight tetrabrominated products with significant yields were quantified, of which three were of unknown substitution pattern and were pseudo quantified against 2′-OH-BDE66 (the response factor between different tetrabrominated congeners differed only marginally on the GC-MS system used). The major tetrabrominated congener formed was 6′-OH-BDE49 followed by an unknown compound (U4-OH-A in Table S4, in the ESM). During chemical debromination, on the other hand, 6′-OH-BDE49 was only formed as a moderate to minor product from 6-OH-BDE90 and 6-OH-BDE99, while 2′-OH-BDE68 and 6-OH-BDE47, respectively, were formed as the major products. The difference in the product composition gained between chemical debromination and debromination in sediment highlights the added selectivity during debromination in nature. Of course, the product composition may vary depending on several abiotic factors such as redox potential and pH, as well as by the composition of bacteria strains in the sediment. The chemical debromination conducted in this study utilized hydrochloric acid, which means that the pH will be much lower than what is usually found in natural sediments.

As the debromination progressed further in the sediment, two tribrominated congeners became utterly dominant. One of these congeners was indicated as a minor product of 6′-OH-BDE49 in the chemical debromination experiment (U3-OH-BDE49 in Table S4, in the ESM) and the other as a minor product of 6-OH-BDE47 (U3-OH-BDE47 in Table S4, in the ESM). However, the large increase of these two congeners in the sediment indicates that some of the unknown tetrabrominated congeners likely degrade to either of these two tribrominated congeners as well (see Table S4, in the ESM). Both these congeners were pseudo quantified against 6′-OH-BDE17. After 7 days in room temperature, the increase in concentration of tetra- and tribrominated congeners accounted for 75% of the decrease in concentration of the added pentabrominated congeners, which proves that debromination is the major degradation route for pentabrominated OH-PBDEs. However, after one month, even the tribrominated congeners started to decrease in concentration (see Fig. 3), while no significant formation of dibrominated congeners was observed. The lack of formed dibrominated congeners indicates that other degradation routes become more important for the less brominated congeners. Representative chromatograms from the experiment can be found in the ESM, Fig. S2.

At 4 °C, no statistically significant degradation of the spiked pentabrominated OH-PBDEs could be determined until after 6 months, at which point 30–50% had been degraded, mainly by debromination (see Fig. 3 as well as Table S5 in the ESM). Only 6-OH-BDE99 reached 50% degradation within 6 months (see Table S5 in the ESM). After one year, 6-OH-BDE90 had reached 50% degradation as well, while 2-OH-BDE123 and 6-OH-BDE85 had decreased in concentration by 39% and 32%, respectively (see Table S5 in the ESM). The increase of the tetra- and tribrominated congeners was more easily measured and a significant increase of some congeners could be observed already after 7 days at 4 °C (see Fig. 3 as well as Table S5 in the ESM).

**Fig. 4:**
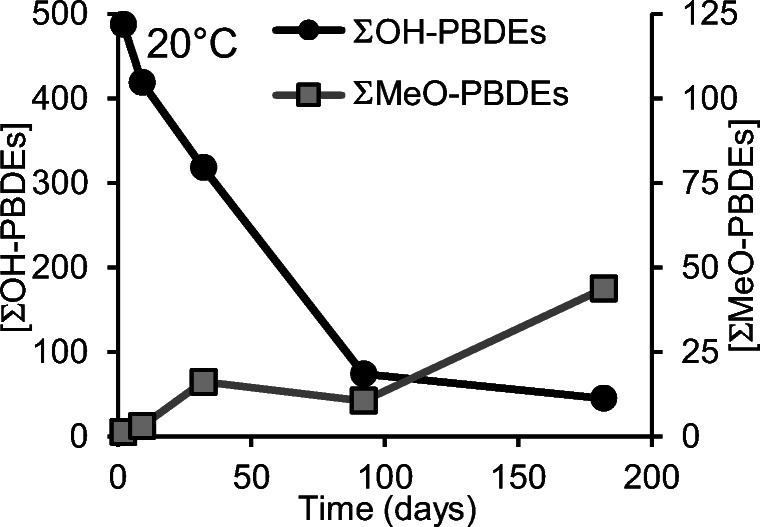
Changes in ΣOH-PBDE, ΣMeO-PBDE concentration over time at room temperature. Concentrations are provided in pmol/g wet weight. Raw data can be found in the ESM Table S4 and S6

During sediment incubation experiments with 6-OH-BDE47, using marine sediment from Liaodong Bay (China), Zhang and co-workers (2012) observed that most of the added compound had been biotransformed to 6-MeO-BDE47 after 66 days at 30 °C. In the present study, methylation of OH-PBDEs was observed to be much slower than debromination (see Fig. 4 as well as Tables S6 and S7 in the ESM). After 6 months at room temperature, the increase of pentabrominated MeO-PBDEs corresponded to 1.5% of the decrease in pentabrominated OH-PBDEs (see Table S6 in the ESM). When adding up all the MeO-PBDEs formed after 6 months in room temperature, they accounted for 9.5% of the degraded pentabrominated OH-PBDEs (see Table S6 in the ESM). At 4 °C, the methylation progressed even slower (see Table S7 in the ESM). The difference in the biotransformations observed between the two studies may be the result of a large difference in the bacterial flora between the marine sediment of Liaodong Bay and the brackish water sediment of the northern Baltic proper.

Although the degradation of OH-PBDEs progressed much slower at 4 °C compared to room temperature, it still seems as if Baltic Sea sediment may have a high capacity for degrading these compounds. The major initial route for degradation seems to be debromination. However, as the OH-PBDEs become less brominated, other degradation routes seem to take over. A small amount of methylation is to be expected in Baltic Sea sediment, although not to the extent as might be observed in, e.g., the marine sediment of Liadong Bay (Zhang et al. 2012). As can be observed in Fig. 4, after 6 months at room temperature, all the determined OH-PBDEs and MeO-PBDEs together only accounted for 20% of the starting concentration, meaning that 80% had been degraded to something that could not be observed using the analytical method applied in this study. Chemical debromination could not be used to predict the degradation products in the sediment. In this study, only surface sediment was used, and it is likely that bacterial activity decreases deeper down in the sediment and thus that the biological degradation of OH-PBDEs slows down in Baltic Sea sediment over time.

### Native occurrence of OH- and MeO-PBDEs in sediment

**Fig. 5:**
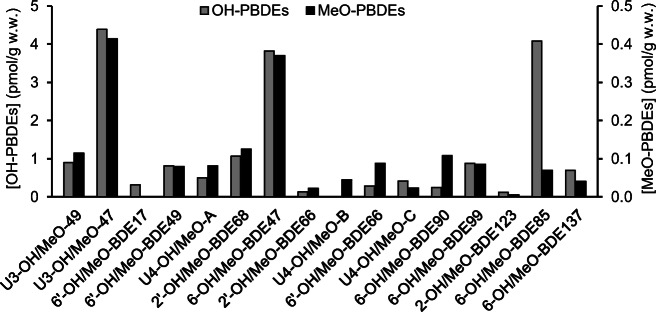
Native occurrence of OH-PBDEs and MeO-PBDEs in Baltic Sea surface sediment. Concentrations are provided in pmol/g wet weight (w.w.). Raw data can be found in the ESM, Table S8

6-OH-BDE85 was by far the most abundant pentabrominated OH-PBDE in the collected Baltic Sea surface sediment (see Fig. 5). The dominance of 6-OH-BDE85 in the sediment is likely a reflection of the large production of this congener observed in Baltic Sea macroalgae, e.g., *C. tenuicorne* (Lindqvist et al. 2017). 6-OH-BDE47, a potential degradation product of 6-OH-BDE85, was the dominant tetrabrominated congener, and its degradation product U3-OH-BDE47 was the dominant tribrominated congener. Among red, green, and brown filamentous macroalgae in the Baltic Sea the OH-PBDE congener pattern is normally dominated by 6-OH-BDE85 and 6-OH-BDE137 shortly followed by 6-OH-BDE47, while tribrominated congeners are rarely observed (Löfstrand 2011; Lindqvist et al. 2017). The levels of MeO-PBDEs were about 10 times lower than that of the corresponding OH-PBDEs (see Fig. 5). Similar relationships between OH- and MeO-PBDEs have also been observed in algae, e.g., *C. tenuicorne* (Dahlgren et al. 2015). Considering the slow conversion from OH-PBDEs to MeO-PBDEs that was observed in the degradation experiment (see Fig. 4), in comparison to debromination (see Fig. 3), a general increase of MeO-PBDEs deeper down in the sediment is not expected.

## Supplementary information

ESM 1 (PDF 419 kb)

## Data Availability

All relevant data are included in the supplementary material.
