# Supplementary material for: Degradation of naturally produced hydroxylated polybrominated diphenyl ethers in Baltic Sea sediment via reductive debromination
Source: Environ Sci Pollut Res Int. 2021 Jan 21;28(20):25878–85. doi: 10.1007/s11356-021-12462-3 (PMC8154838; doi:10.1007/s11356-021-12462-3)
Supplement: Supplementary file 1 — (PDF 419 kb) [file 11356_2021_12462_MOESM1_ESM.pdf]

Electronic Supplementary Material

Degradation of naturally produced hydroxylated polybrominated diphenyl ethers in Baltic Sea sediment via reductive debromination

Dennis Lindqvist[1,*], Johan Gustafsson[1]

[1]Department of Environmental science, Stockholm University, SE-106 91 Stockholm, Sweden

[*]Corresponding author: Telephone: +46 8 163995, e-mail: dennis.lindqvist@aces.su.se,

ORCID: https://orcid.org/0000-0003-0178-2801

11 pages including 8 tables (Table S1-S8) and 2 figures (Figure S1-S2).

Additional results and raw data

Chemical reductive debromination

Table S1 Chemical debromination of individual OH-PBDEs and BDE99. Concentrations provided as relative to the starting concentration set as 1. $T_{1/2}$ was calculated from the slope given by plotting ln[C] vs. time (see Figure 1 in the main article).

	**A**	**B**	**C**		**A**	**B**	**C**		**A**	**B**	**C**		**A**	**B**	**C**
Time (min)	6-OH-BDE137			T	6-OH-BDE85			T	2-OH-BDE123			T	6-OH-BDE90		
**0**	1	1	1	**0**	1	1	1	**0**	1	1	1	**0**	1	1	1
**1**	0.76	0.52	0.53	**2**	0.43	0.49	0.52	**2**	0.46	0.53	0.54	**2**	0.51	0.58	0.60
**3**	0.51	0.36	0.35	**5**	0.25	0.30	0.36	**5**	0.27	0.36	0.40	**5**	0.33	0.42	0.45
**6**	0.26	0.19	0.19	**9**	0.10	0.18	0.18	**9**	0.11	0.20	0.19	**9**	0.16	0.26	0.25
**10**	0.11	0.08	0.08	**14**	0.03	0.08	0.06	**14**	0.03	0.09	0.07	**14**	0.06	0.15	0.11
**15**	0.03	0.03	0.02	**20**	0.01	0.02	0.02	**20**	0.01	0.03	0.02	**20**	0.03	0.06	0.05
$t_{1/2}$ (min)	3.1	3.1	3.0		2.9	3.9	3.7		3.1	4.0	3.7		3.9	5.1	4.6
	6-OH-BDE99				6-MeO-BDE99				BDE99				2'-OH-BDE66		
**0**	1	1	1	**0**	1	1	1								
**2**	0.57	0.68	0.68	**2**	0.59	0.67	0.67	**0**	1	1	1	**0**	1	1	1
**5**	0.39	0.50	0.53	**5**	0.41	0.48	0.45	**5**	0.66	0.62	0.61	**15**	0.52	0.65	0.56
**9**	0.20	0.32	0.32	**9**	0.23	0.28	0.26	**15**	0.28	0.26	0.26	**45**	0.19	0.28	0.22
**14**	0.09	0.19	0.15	**14**	0.11	0.12	0.12	**30**	0.09	0.09	0.09	**90**	0.04	0.09	0.07
**20**	0.04	0.08	0.07	**20**	0.03	0.04	0.04	**60**	0.02	0.01	0.01	**150**	0.01	0.02	0.01
$t_{1/2}$ (min)	4.3	5.7	5.2		4.3	4.6	4.5		10	9.7	9.5		20	24	27
	2'-OH-BDE68				6'-OH-BDE49				6-OH-BDE47						
**0**	1	1	1	**0**	1	1	1	**0**	1	1	1				
**15**	0.96	1.01	0.94	**45**	0.87	1.07	0.86	**45**	0.88	0.87	1.07				
**45**	0.74	0.77	0.72	**90**	0.79	0.85	0.80	**90**	0.81	0.82	0.89				
**90**	0.50	0.59	0.61	**180**	0.71	0.72	0.70	**180**	0.72	0.71	0.88				
**150**	0.41	0.47	0.43	**240**	0.64	0.69	0.65	**240**	0.66	0.66	0.74				
$t_{1/2}$ (min)	122	136	108		408	277	408		433	433	385				

Grey highlighted values were not included in the calculation of $t_{1/2}$.

Table S2 Formed products during chemical debromination of OH-PBDEs. The relative formation of the different products is provided only as major, moderate, or minor at 30-70% degradation of the parent compound. The values refer to the retention time on the GC-MS.

	**6-OH-BDE90**	17.06		**6-OH-BDE99**	17.17		**2-OH-BDE123**	17.98		**6-OH-BDE85**	18.43	
OH-tetraBDEs				2'-OH-BDE66	15.47	Minor	6'-OH-BDE66	15.66	Major	6'-OH-BDE66	15.66	Major
	Unknown	15.74	Minor	6-OH-BDE47	14.92	Major	Unknown	15.56	Minor	Unknown	15.56	Minor
	2'-OH-BDE68	14.29	Major	Unknown	14.72	Minor	2'-OH-BDE66	15.47	Minor	6-OH-BDE47	14.92	Major
	6'-OH-BDE49	14.07	Mod/Min	6'-OH-BDE49	14.07	Moderate	2'-OH-BDE68	14.29	Major	Unknown	14.72	Minor
OH-triBDEs	Unknown	13.18	Minor	Unk (2'-66)	12.99	Minor	Unknown	13.25	Mod/Min	Unknown	13.26	Mod/Min
	Unk (2'-68)	12.67	Minor	2'-OH-BDE28	12.83	Mod/Min	Unk (2'-66)	12.99	Minor	2'-OH-BDE28	12.83	Mod/Min
	2'-OH-BDE25	12.26	Mod/Min	Unk (2'-66)	12.76	Minor	2'-OH-BDE28	12.83	Minor	Unknown	12.76	Minor
	Unk (2'-68)	12.18	Moderate	Unk (6-47)	12.63	Mod/Min	Unk (2'-66)	12.76	Minor	Unk (6-47)	12.63	Minor
	Unk (6'-49)	11.99	Minor	Unknown	12.56	Minor	Unk (2'-68)	12.67	Minor	Unknown	12.56	Minor
	Unknown	11.86	Minor	6'-OH-BDE17	12.49	Minor	2'-OH-BDE25	12.26	Minor	Unknown	12.50	Minor
	U3-OH-BDE49	11.71	Minor	Unknown	12.42	Minor	Unk (2'-68)	12.18	Mod/Min	U3-OH-BDE47	12.33	Minor
				U3-OH-BDE47	12.33	Minor	Unk (2'-68)	11.86	Minor			
				2'-OH-BDE25	12.26	Minor	Unknown	11.77	Minor			
				Unk (6'-49)	11.99	Minor						
				U3-OH-BDE49	11.72	Minor						

	**6'-OH-BDE49**	14.07		**2'-OH-BDE68**	14.29		**6-OH-BDE47**	14.91		**2'-OH-BDE66**	15.50	
OH-triBDEs	6'-OH-BDE17	12.50	Minor	Unknown	12.67	Moderate	2'-OH-BDE28	12.83	Major	Unknown	12.99	Minor
	2'-OH-BDE25	12.26	Minor	2'-OH-BDE25	12.26	Minor	Unknown	12.63	Major	Unknown	12.83	Major
	Unknown	12.00	Major	Unknown	12.18	Major	6'-OH-BDE17	12.49	Minor	Unknown	12.76	Minor
	U3-OH-BDE49	11.72	Minor	Unknown	11.86	Minor	U3-OH-BDE47	12.33	Minor	2'-OH-BDE25	12.26	Minor
OH-diBDEs	Unknown	10.79	Minor	Unknown	11.33	Minor	Unknown	10.99	Minor	Unknown	10.99	Minor
	Unknown	10.65	Minor	Unknown	10.94	Minor	Unknown	10.81	Minor	Unknown	10.81	Minor
	Unknown	10.58	Minor	Unknown	10.58	Minor	Unknown	10.79	Minor	Unknown	10.64	Minor
	Unknown	10.05	Minor	Unknown	10.15	Minor	Unknown	10.64	Minor	Unknown	10.58	Minor
							Unknown	10.45	Minor			

2'-OH-BDE25 was tentatively identified as it is the only tribrominated congener that can be formed by 6'-OH-BDE49, 2'-OH-BDE68, and 2'-OH-BDE66.
U3-OH-BDE49 should, if no rearrangements occur, be either 6-OH-BDE18 or 6-OH-BDE31.
U3-OH-BDE47 should, if no rearrangements occur, be either 6-OH-BDE17 or 6-OH-BDE28.
Unk (No.) Indicates an unknown tribrominated congener and which tetrabrominated congener it can be formed from.

Table S3 All potential tetrabrominated congeners formed by removal of one bromine from each of the four pentabrominated congeners tested. Ranked from 1 to 5 in expected relative formation based on calculated energies (kJ/mol) using the MMFF94 forced field to optimize the geometry. Calculated using the Avogadro Software (Hanwell et al. 2012).

		**6-OH-BDE90**	**6-OH-BDE99**	**2-OH-BDE123**	**6-OH-BDE85**
	**kJ/mol**	248	256	252	244
6-OH-BDE47	191.8		1		1
2'-OH-BDE68	192.1	1		1	
6'-OH-BDE66	200			2	2
6-OH-BDE42	237.5	3			4
6'-OH-BDE49	253	4	3		
2'-OH-BDE66	255		4	4	
6-OH-BDE60	233				3
6-OH-BDE63	236.8	2			
2-OH-BDE81	241			3	
6-OH-BDE74	245		2		
6-OH-BDE41	269				5
6-OH-BDE43	274	5			
2-OH-BDE76	277			5	
6-OH-BDE48	282		5		

The first section shows potential congeners formed by removal of one Br from the phenol ring. The second section shows potential congeners formed by removal of one Br from the phenoxy ring. Grey highlighted congeners represent the expected dominant congeners based on significant lower calculated energies compared to the parent compound. Congeners written in *italic* were not available as standards for identification in this study.

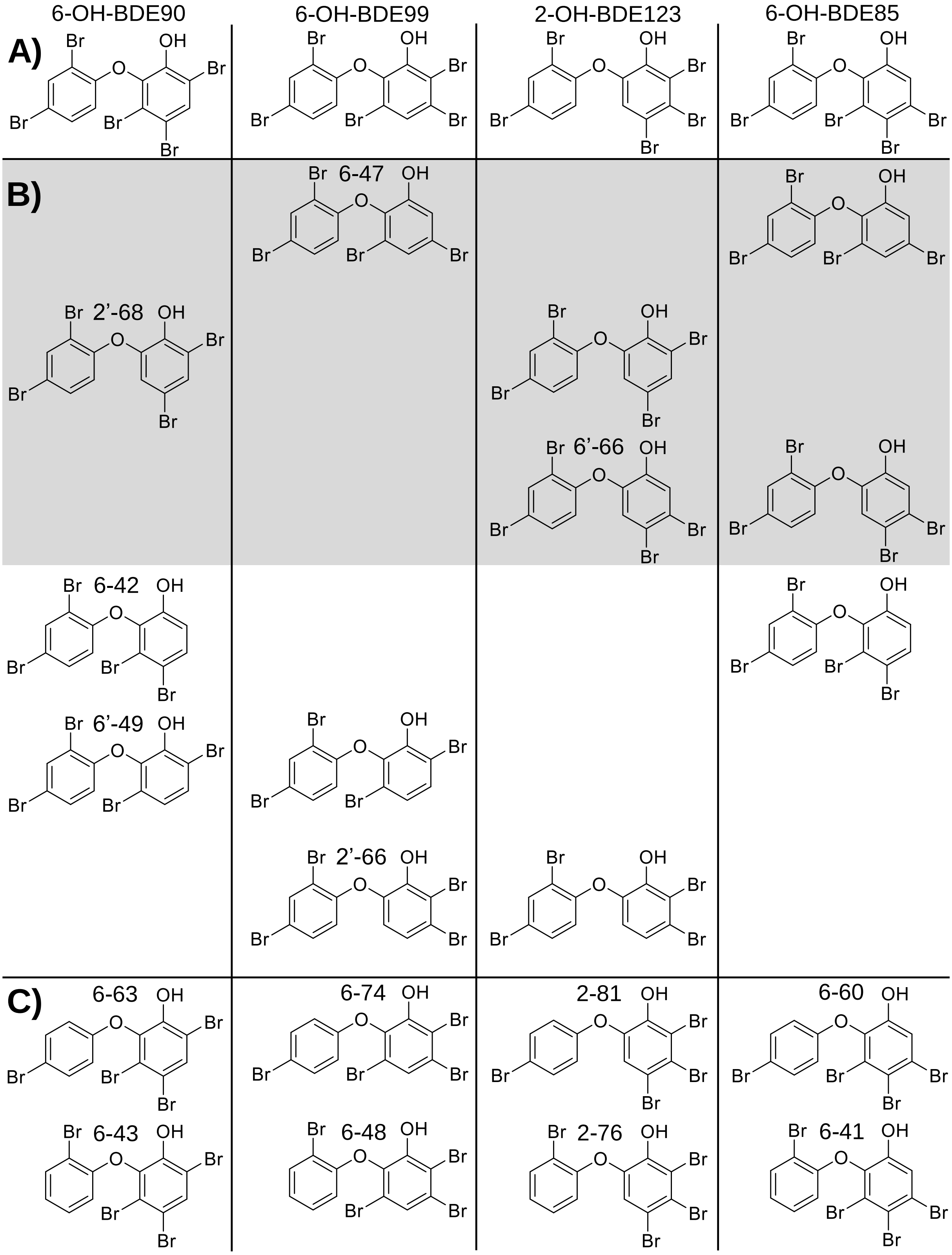

Figure S1 A) Structures of the four pentabrominated congeners degraded in this study. B) Structures of the tetrabrominade congeners that can be formed by removal of one Br from the phenol ring. C) Structures of the tetrabrominade congeners that can be formed by removal of one Br from the phenoxy ring. The structures are ordered according to the order in Table S3

Table S4 Degradation of spiked pentabrominated OH-PBDEs in sediment over time at room temperature, as well as formation of some tetrabrominated- and tribrominated OH-PBDEs. Concentrations provided in pmol/g wet weight.

**Day**	U3-OH-BDE49[A]	U3-OH-BDE47[A]	6'-OH-BDE17	6'-OH-BDE49	U4-OH-A[B]	2'-OH-BDE68	6-OH-BDE47	U4-OH-B[B]	2'-OH-BDE66	6'-OH-BDE66[B]	U4-OH-C[B]	**6-OH-BDE90**	**6-OH-BDE99**	**2-OH-BDE123**	**6-OH-BDE85**
0 A	0.21	1.04	0.05	0.42	0.39	0.91	1.70	0.05	0.12	0.48	0.15	129	138	125	164
0 B	0.16	0.73	0.05	0.48	0.59	0.76	1.32	0.07	0.10	0.26	0.17	89.0	96.0	83.6	109
0 C	0.23	0.66	0.63	2.29	3.82	1.22	2.29	0.44	0.43	0.41	0.46	121	133	113	138
0 Av.	0.20	0.81	0.24	1.06	1.60	0.96	1.77	0.19	0.22	0.38	0.26	113	122	107	137
7 D	56.4	3.34	0.18	68.8	35.7	20.2	5.25	4.40	5.86	0.93	11.9	25.9	38.8	59.3	95.9
7 E	89.1	13.9	0.80	47.8	41.8	21.1	8.95	9.56	4.56	1.30	16.1	29.2	43.4	59.2	89.8
7 F	67.4	23.7	1.76	25.9	23.4	5.76	10.3	8.55	4.44	1.50	12.1	25.7	28.9	35.9	72.5
7 Av.	70.9	13.7	0.91	47.5	33.6	15.7	8.16	7.50	4.95	1.24	13.4	26.9	37.0	51.4	86.1
30 G	100	46.3	0.52	14.2	15.0	14.3	7.05	5.81	4.53	1.03	18.5	16.1	13.4	27.5	42.3
30 H	5.71	3.84	0.16	9.40	5.53	2.10	3.76	1.60	1.09	0.53	1.71	24.3	24.5	27.1	36.8
30 I	71.1	56.8	1.63	28.7	12.6	12.5	14.3	2.79	3.30	1.81	15.1	53.6	54.3	50.5	96.3
30 Av.	59.1	35.7	0.77	17.4	11.1	9.61	8.38	3.40	2.97	1.12	11.8	31.3	30.7	35.0	59.8
90 J	5.71	2.86	0.08	2.38	1.79	1.05	1.41	0.56	0.33	0.18	1.08	9.86	8.87	10.6	15.4
90 K	8.95	7.02	0.12	3.71	4.51	2.72	4.10	1.44	0.63	0.79	4.25	12.2	10.2	15.0	28.7
90 L	4.04	2.50	0.09	1.70	1.65	0.82	1.38	0.49	0.23	0.14	1.00	9.53	8.33	9.12	14.8
90 Av.	6.23	4.13	0.10	2.60	2.65	1.53	2.30	0.83	0.40	0.37	2.11	10.5	9.12	11.6	19.7
180 M	1.49	1.87	0.07	1.48	1.08	1.04	3.72	0.37	0.22	0.99	1.06	5.29	4.23	5.51	11.6
180 N	2.27	1.25	0.08	2.16	1.09	1.20	2.53	0.33	0.33	0.57	0.51	5.00	4.16	5.26	10.2
180 O	1.51	3.13	0.09	1.77	1.46	2.00	9.56	0.45	0.26	2.39	0.90	6.68	5.45	6.20	16.4
180 Av.	1.76	2.08	0.08	1.80	1.21	1.41	5.27	0.38	0.27	1.32	0.82	5.65	4.61	5.65	12.7

[A] Pseudo quantified against 6'-OH-BDE17.
[B] Pseudo quantified against 2'-OH-BDE66.

Table S5 Degradation of spiked pentabrominated OH-PBDEs in sediment over time at 4 °C, as well as formation of some tetrabrominated- and tribrominated OH-PBDEs. Concentrations provided in pmol/g wet weight.

**Day**	U3-OH-BDE49[A]	U3-OH-BDE47[A]	6'-OH-BDE17	6'-OH-BDE49	U4-OH-A[B]	2'-OH-BDE68	6-OH-BDE47	U4-OH-B[B]	2'-OH-BDE66	6'-OH-BDE66[B]	U4-OH-C[B]	**6-OH-BDE90**	**6-OH-BDE99**	**2-OH-BDE123**	**6-OH-BDE85**
0 A	0.21	1.04	0.05	0.42	0.39	0.91	1.70	0.05	0.12	0.48	0.15	129	138	125	164
0 B	0.16	0.73	0.05	0.48	0.59	0.76	1.32	0.07	0.10	0.26	0.17	89.0	96.0	83.6	109
0 C	0.23	0.66	0.63	2.29	3.82	1.22	2.29	0.44	0.43	0.41	0.46	121	133	113	138
0 Av.	0.20	0.81	0.24	1.06	1.60	0.96	1.77	0.19	0.22	0.38	0.26	113	122	107	137
7 D	1.38	0.86	0.05	5.75	11.7	1.32	2.69	1.23	0.91	0.37	0.95	87.5	90.2	75.8	98.4
7 E	0.20	0.77	0.04	0.84	1.71	1.22	2.04	0.15	0.12	0.37	0.26	111	116	95.3	122
7 F	2.15	0.94	0.04	9.55	13.6	2.07	2.92	1.57	1.24	0.45	1.33	101	109	95.2	122
7 Av.	1.24	0.86	0.04	5.38	9.03	1.54	2.55	0.98	0.76	0.40	0.84	100	105	88.8	114
30 G	0.17	0.65	0.03	0.87	1.36	1.67	1.95	0.12	0.09	0.39	0.27	126	115	120	152
30 H	13.8	2.06	0.29	31.7	15.2	6.11	4.98	3.22	2.86	0.68	2.47	67.6	77.2	76.3	101
30 I	26.1	7.84	0.32	22.6	25.5	7.31	7.13	3.61	2.65	0.90	4.12	102	106	98.5	131
30 Av.	13.3	3.52	0.21	18.4	14.0	5.03	4.69	2.32	1.87	0.66	2.29	98.7	99.4	98.4	128
180 J	36.7	10.4	0.90	25.8	20.5	10.5	12.4	4.63	3.78	2.20	4.80	49.8	51.6	57.1	84.7
180 K	0.43	3.00	0.04	6.04	7.52	12.9	12.5	0.86	0.22	3.00	1.24	96.8	79.2	85.8	119
180 L	57.3	20.4	1.33	27.7	27.3	10.2	15.2	5.22	4.15	3.63	5.65	52.4	50.5	55.0	82.5
180 Av.	31.5	11.3	0.76	19.8	18.4	11.2	13.4	3.57	2.72	2.94	3.89	66.3	60.4	66.0	95.4
360 M	14.0	6.56	0.06	19.4	32.2	12.4	10.0	5.52	1.03	1.42	6.88	67.8	62.4	79.0	109
360 N	35.3	24.2	0.75	13.7	18.8	9.27	14.8	3.69	2.74	3.20	5.86	40.9	40.8	47.6	74.1
360 O	22.6	10.7	0.18	17.8	33.2	13.1	13.9	6.04	1.66	2.42	6.92	61.0	59.6	70.3	96.3
360 Av.	24.0	13.8	0.3	16.9	28.1	11.6	12.9	5.1	1.8	2.3	6.6	56.6	54.2	65.7	93.3

[A] Pseudo quantified against 6'-OH-BDE17.
[B] Pseudo quantified against 2'-OH-BDE66.

Table S6 Formation of MeO-PBDEs in sediment over time at room temperature. Concentrations provided in pmol/g wet weight.

**Day**	U3-MeO-BDE49[A]	U3-MeO-BDE47[A]	6'-OH-BDE17	6'-MeO-BDE49	U4-MeO-A[B]	2'-MeO-BDE68	6-MeO-BDE47	U4-MeO-B[B]	2'-MeO-BDE66	6'-MeO-BDE66[B]	U4-MeO-C[B]	6-MeO-BDE90	6-MeO-BDE99	2-MeO-BDE123	6-MeO-BDE85
0 A	0.06	0.17	<LOQ	0.03	0.10	0.08	0.19	0.08	<LOQ	NQ	0.05	0.23	0.16	0.10	0.05
0 B	0.07	0.22	<LOQ	0.03	0.02	0.10	0.24	0.02	<LOQ	NQ	0.02	0.32	0.15	0.11	0.06
0 C	0.05	0.17	<LOQ	0.04	0.01	0.07	0.17	0.02	<LOQ	NQ	0.02	0.32	0.18	0.12	0.06
0 Av.	0.06	0.19	<LOQ	0.03	0.05	0.08	0.20	0.04	<LOQ	NQ	0.03	0.29	0.16	0.11	0.05
7 D	0.05	0.10	<LOQ	0.03	0.05	0.07	0.11	0.04	<LOQ	NQ	0.02	0.26	0.16	0.17	0.05
7 E	0.23	0.15	<LOQ	0.18	0.21	0.10	0.13	0.07	<LOQ	NQ	0.03	0.36	0.26	0.20	0.07
7 F	2.78	0.22	<LOQ	0.61	0.89	0.13	0.14	0.17	<LOQ	NQ	0.05	0.49	0.44	0.33	0.08
7 Av.	1.02	0.15	<LOQ	0.27	0.38	0.10	0.13	0.09	<LOQ	NQ	0.03	0.37	0.29	0.23	0.07
30 G	10.9	NQ	<LOQ	0.69	1.39	0.46	0.23	0.40	0.25	NQ	0.70	0.41	NQ	0.85	0.49
30 H	2.87	0.45	<LOQ	0.90	1.30	0.30	0.23	0.40	0.07	NQ	0.11	0.84	0.87	0.88	0.26
30 I	14.0	0.41	<LOQ	NQ	2.02	1.26	0.14	0.34	0.30	NQ	0.10	0.41	0.81	0.94	0.13
30 Av.	9.25	0.43	<LOQ	0.79	1.34	0.67	0.20	0.38	0.21	NQ	0.31	0.55	0.84	0.89	0.29
90 J	4.85	2.92	<LOQ	0.83	1.07	0.41	0.50	0.44	0.06	NQ	0.82	0.94	0.75	1.68	2.39
90 K	1.44	0.48	<LOQ	0.56	0.35	0.35	0.49	0.10	0.10	NQ	0.08	0.52	0.45	0.44	0.43
90 L	2.86	1.32	<LOQ	0.47	0.64	0.29	0.07	0.29	0.04	NQ	0.41	0.39	0.39	0.63	0.42
90 Av.	3.05	1.57	<LOQ	0.62	0.68	0.35	0.35	0.28	0.07	NQ	0.44	0.61	0.53	0.91	1.08
180 M	1.23	1.26	<LOQ	0.65	0.68	0.37	0.73	0.22	0.16	NQ	NQ	0.67	0.49	0.59	0.38
180 N	28.9	26.8	<LOQ	4.38	10.1	4.51	3.17	4.67	0.76	NQ	8.30	3.01	1.81	6.75	4.84
180 O	1.40	1.82	<LOQ	1.12	0.95	0.90	1.55	0.24	0.31	NQ	0.21	1.22	0.78	0.64	0.55
180 Av.	10.5	10.0	<LOQ	2.05	3.92	1.93	1.82	1.71	0.41	NQ	4.25	1.63	1.03	2.66	1.93

[A] Pseudo quantified against 6'-MeO-BDE17.
[B] Pseudo quantified against 2'-MeO-BDE66.
<LOQ = Detected, but at very low levels, below the limit of quantification.
NQ = Detected but not quantifiable due to distorted peak and/or co-eluting peaks.

Table S7 Formation of MeO-PBDEs in sediment over time at 4 °C. Concentrations provided in pmol/g wet weight.

**Day**	U3-MeO-BDE49[A]	U3-MeO-BDE47[A]	6'-OH-BDE17	6'-MeO-BDE49	U4-MeO-A[B]	2'-MeO-BDE68	6-MeO-BDE47	U4-MeO-B[B]	2'-MeO-BDE66	6'-MeO-BDE66[B]	U4-MeO-C[B]	6-MeO-BDE90	6-MeO-BDE99	2-MeO-BDE123	6-MeO-BDE85
0 A	0.06	0.17	<LOQ	0.03	0.10	0.08	0.19	0.08	<LOQ	NQ	0.05	0.23	0.16	0.10	0.05
0 B	0.07	0.22	<LOQ	0.03	0.02	0.10	0.24	0.02	<LOQ	NQ	0.02	0.32	0.15	0.11	0.06
0 C	0.05	0.17	<LOQ	0.04	0.01	0.07	0.17	0.02	<LOQ	NQ	0.02	0.32	0.18	0.12	0.06
0 Av.	0.06	0.19	<LOQ	0.03	0.05	0.08	0.20	0.04	<LOQ	NQ	0.03	0.29	0.16	0.11	0.05
180 D	3.87	0.63	0.06	1.35	1.77	0.48	0.37	0.41	0.53	<LOQ	<LOQ	1.19	0.81	0.72	0.19
180 E	0.17	0.37	0.03	0.15	0.13	0.21	0.34	<LOQ	<LOQ	0.36	<LOQ	0.49	0.25	0.22	0.09
180 F	5.81	0.83	0.09	1.43	1.69	0.44	0.46	0.39	0.47	0.41	0.09	1.19	0.79	0.72	0.27
180 Av.	3.28	0.61	0.06	0.97	1.20	0.38	0.39	0.40	0.50	0.39	0.09	0.96	0.62	0.55	0.18
360 G	0.35	0.24	0.02	0.60	0.74	0.20	0.20	0.23	0.03	NQ	0.06	0.66	0.36	0.59	0.12
360 H	4.92	0.83	0.06	1.17	2.16	0.44	0.36	0.35	0.42	0.21	<LOQ	1.16	0.86	0.74	0.24
360 I	10.2	1.76	0.04	4.79	9.14	2.31	1.13	1.15	0.23	0.32	0.41	3.98	2.32	3.35	1.22
360 Av.	5.15	0.95	0.04	2.19	4.01	0.98	0.56	0.58	0.23	0.26	0.24	1.93	1.18	1.56	0.53

[A] Pseudo quantified against 6'-MeO-BDE17.
[B] Pseudo quantified against 2'-MeO-BDE66.
<LOQ = Detected, but at very low levels, below the limit of quantification.
NQ = Detected but not quantifiable due to distorted peak and/or co-eluting peaks.
The samples taken after 7 and 30 days respectivelly were not quantified for MeO-PBDEs as no significant changes had occured after such short time.

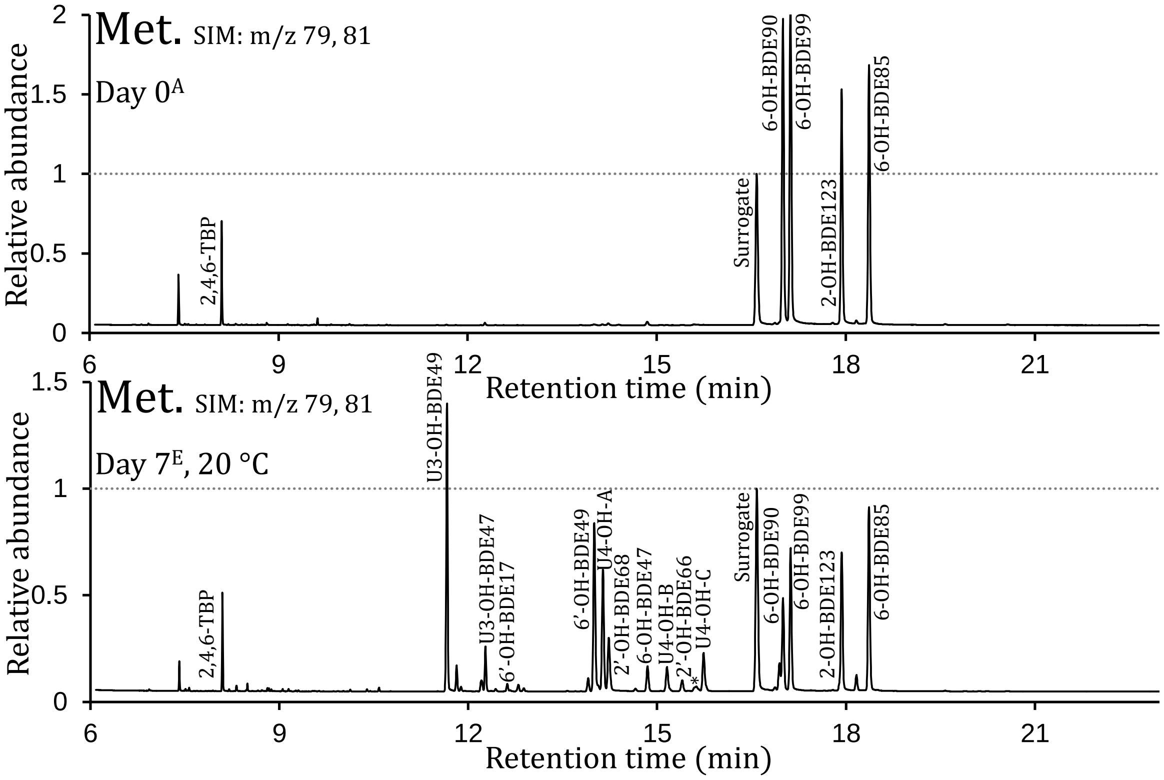

Figure S2 GC-MS SIM chromatograms of methylated samples. Above: Day 0 (sample A, see Table S4). Below: Day 7 in room temperature (sample E, see Table S4). Peak intensity is given as relative abundance to that of the surrogate standard (set to 1). *6'-OH-BDE66.

Native occurrence of OH- and MeO-PBDEs in sediment

Table S8 Native concentration of OH-PBDEs and MeO-PBDEs in Baltic Sea sediment provided in pmol/g wet weight.

	Phenolic		Neutral	
	A	B	A	B
U3-OH/MeO-BDE49[A]	0.77	1.03	0.11	0.12
U3-OH/MeO-BDE47[A]	3.67	5.10	0.42	0.41
6'-OH/MeO-BDE17	0.27	0.35	ND	ND
2'-OH/MeO-BDE28	NQ	NQ	ND	ND
6'-OH/MeO-BDE49	0.67	0.96	0.08	0.08
U4-OH/MeO-A[B]	0.42	0.59	0.08	0.08
2'-OH/MeO-BDE68	0.95	1.18	0.13	0.12
6-OH/MeO-BDE47	3.15	4.49	0.37	0.37
2'-OH/MeO-BDE66	0.11	0.15	0.03	0.02
U4-OH/MeO-B[B]	ND	ND	0.05	0.04
6'-OH/MeO-BDE66[B]	0.24	0.33	0.09	0.08
U4-OH/MeO-C[B]	0.35	0.49	0.02	0.02
6-OH/MeO-BDE90	0.21	0.29	0.12	0.10
6-OH/MeO-BDE99	0.72	1.0	0.08	0.09
2-OH/MeO-BDE123	0.10	0.15	0.01	0.01
6-OH/MeO-BDE85	3.30	4.86	0.07	0.07
6-OH/MeO-BDE137	0.56	0.83	0.04	0.04
Recovery SS	72%	79%	NA	NA

[A] Pseudo quantified against 6'-OH/MeO-BDE17.
[B] Pseudo quantified against 2'-OH/MeO-BDE66.
ND = Not detected.
NQ = Not quantifiable due to distorted peak.
NA = Not analyzed

References

Hanwell MD, Curtis DE, Lonie DC, Vandermeersch T, Zurek E, Hutchison GR (2012) Avogadro: An advanced semantic chemical editor, visualization, and analysis platform. Journal of Cheminformatics 4:17.

Avogadro: an open-source molecular builder and visualization tool. Version 1.2.0 http://avogadro.cc/
